# Supplementary material for: Reagent-Free Immobilization of Industrial Lipases to Develop Lipolytic Membranes with Self-Cleaning Surfaces
Source: Membranes (Basel). 2022 Jun 9;12(6):599. doi: 10.3390/membranes12060599 (PMC9229154; doi:10.3390/membranes12060599)
Supplement: Supplementary file 1 [file membranes-12-00599-s001.zip › membranes-1742276-supplementary.pdf]

membranes

*Supplementary Materials*

# Reagent-Free Immobilization of Industrial Lipases to Develop Lipolytic Membranes with Self-Cleaning Surfaces

**Martin Schmidt [1], Andrea Prager [1], Nadja Schönherr [1], Roger Gläser [2] and Agnes Schulze [1,*]**

[1] Leibniz Institute of Surface Engineering (IOM), Permoserstr. 15, 04318 Leipzig, Germany; martin.schmidt@iom-leipzig.de (M.S.); andrea.prager@iom-leipzig.de (A.P.); nadja.schoenherr@iom-leipzig.de (N.S.)
[2] Institute of Chemical Technology, Leipzig University, Linnéstraße 3, 04103 Leipzig, Germany; roger.glaeser@uni-leipzig.de
* Correspondence: agnes.schulze@iom-leipzig.de

**Keywords:** enzyme membrane reactor; lipase; fouling; self-cleaning surface; electron beam; response surface methodology

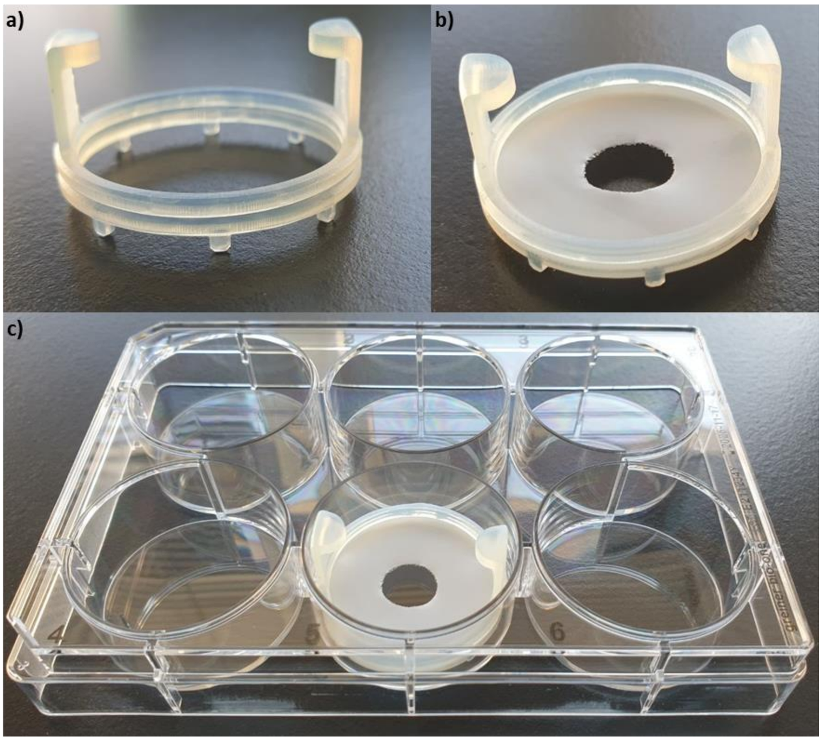


**Figure S1**. Implementation of the kinetic assay. For the tests, (**a**) a 3D-printed scaffold was used, into which (**b**) a membrane sample with a centered hole was inserted, and (**c**) placed into a 6-well plate. Finally, the chromogenic substrate solution was added and the release of the dye 4-nitrophenol was continuously measured photometrically.

```
[task]
   task = fit
   data = generic
[parameters]
   t, So
   Vmax, Km
   D
[model] ;units: μM, s, μM/s
   So = 200
   Km = 10 ??
   Vmax = 1 ??
   D = 0.0001 ? (-1 .. +1)
   P = So - Km*W(So/Km*exp((So - Vmax*t)/Km)) + D*t
[data]
   variable t
   directory ./projects/lipase/data
   sheet data.csv
   column 2
[output]
   directory ./projects/lipase/output/
[settings]
{ConfidenceIntervals}
   SquaresIncreasePercent = 10
{Filter}
   XMin = 30
{Output}
   XAxisLabel = t, sec
   YAxisLabel = c, uM
[end]
```

**Figure S2**. DynaFit script. The integrated closed-form Michaelis-Menten rate equation was used according to the manuscript. Please note that a correction term, *D*, was added to account for non-specific effects such as adsorption of the dye or substrate, product inhibition, *etc*.

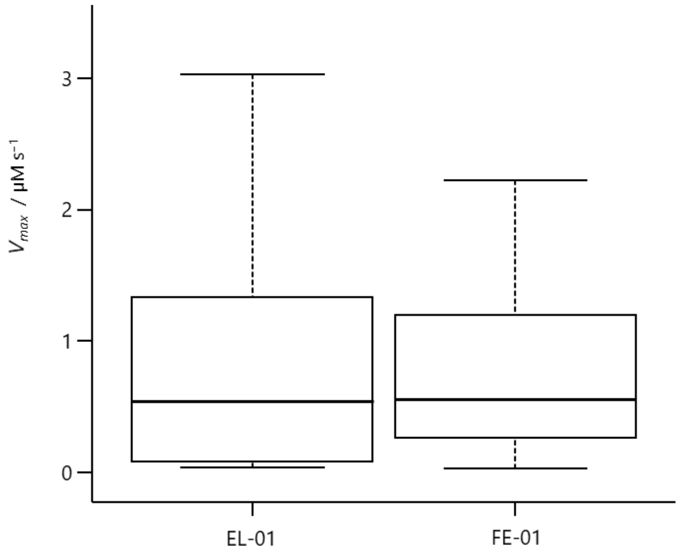


**Figure S3**. Boxplots of reaction rates. Both enzymes, EL-01 and FE-01, give the same average $V_{max}$, with EL-01 showing slightly higher maximum values.

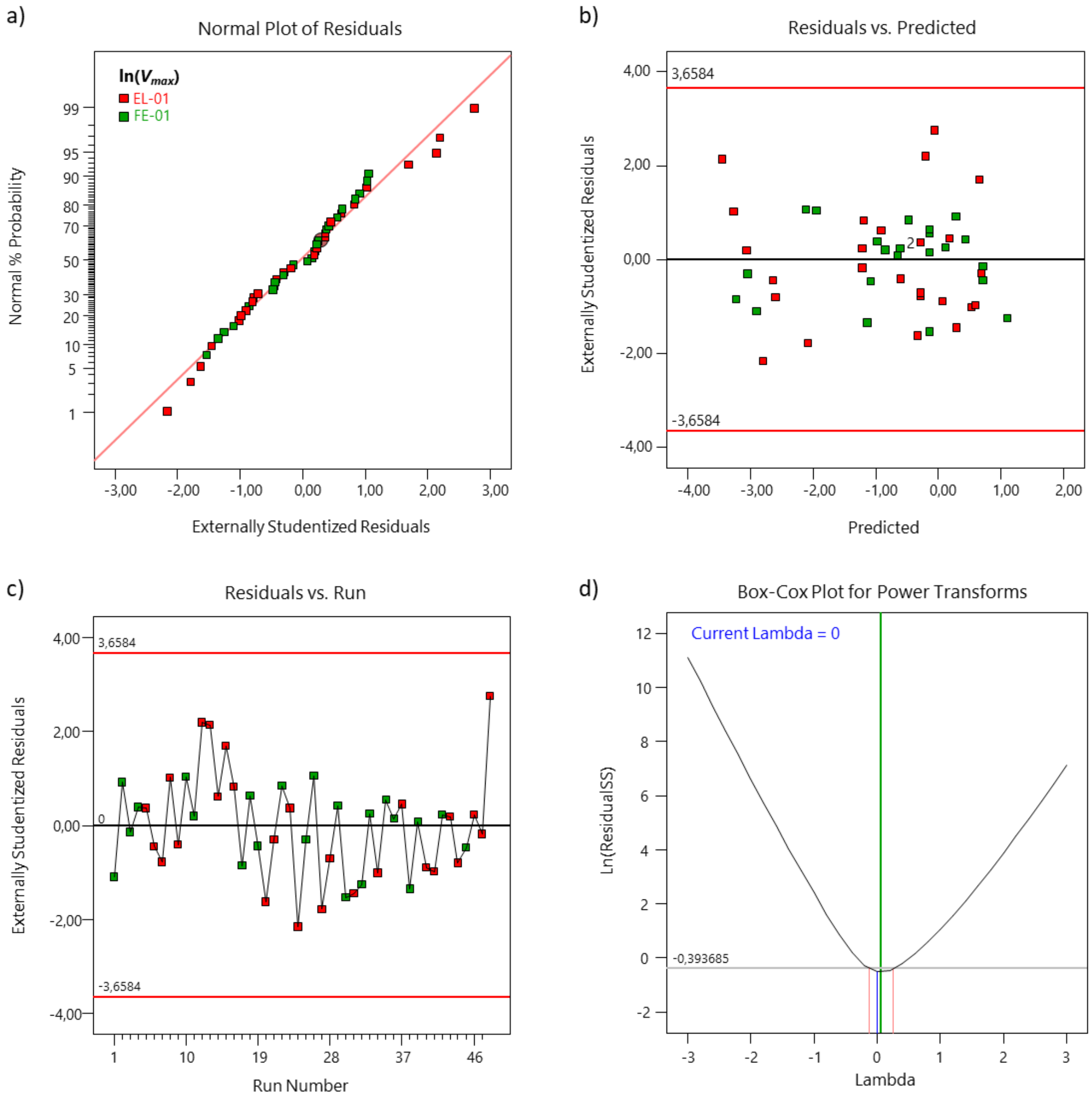


**Figure S4**. Diagnostics of RSM design. The final model was supported by several diagnostics in Design-Expert 13, *e.g.*, (**a**) test for normal distribution with normal probability plot; (**b**) test for homoscedasticity with residual plot; (**c**) test for randomness with residual-run plot; and (**d**) test for data transformation with Box-Cox plot (all after *ln* transformation).

**Table S1**. Data of RSM design. Given are the factor settings per run an the responses (kinetic parameters) obtained with the DynaFit software package (*cf.* manuscript and Figure S2).

| Run | Factors | | | | Responses | | | |
|---|---|---|---|---|---|---|---|---|
| | **A: conc. / g L$^{-1}$** | **B: time / min** | **C: dose / kGy** | **D: enzyme** | $V_{max}$ **/ µM s$^{-1}$** | $K_m$ **/ µM** | $V/K$ **/ 10$^{-3}$ s$^{-1}$** | $D$ **/ µM s$^{-1}$** |
| 1 | 1.0 | 5.1 | 125 | FE-01 | 0.04 | 94.3 | 0.5 | -0.0048 |
| 2 | 8.2 | 2.1 | 80 | FE-01 | 1.71 | 86.4 | 19.8 | -0.0603 |
| 3 | 8.2 | 8.0 | 80 | FE-01 | 1.97 | 84.9 | 23.2 | -0.0288 |
| 4 | 2.8 | 2.1 | 80 | FE-01 | 0.42 | 61.9 | 6.8 | -0.0141 |
| 5 | 5.5 | 5.1 | 125 | EL-01 | 0.85 | 31.2 | 27.1 | -0.0297 |
| 6 | 1.0 | 5.1 | 125 | EL-01 | 0.06 | 45.9 | 1.4 | -0.0056 |
| 7 | 5.5 | 5.1 | 125 | EL-01 | 0.59 | 28.1 | 21.0 | -0.0297 |
| 8 | 2.8 | 8.0 | 170 | EL-01 | 0.05 | 56.3 | 0.9 | -0.0047 |
| 9 | 2.8 | 2.1 | 50 | EL-01 | 0.50 | 61.5 | 8.2 | -0.0306 |
| 10 | 5.5 | 0.1 | 125 | FE-01 | 0.19 | 58.6 | 3.2 | -0.0186 |
| 11 | 2.8 | 8.0 | 50 | FE-01 | 0.45 | 69.4 | 6.5 | -0.0220 |
| 12 | 2.8 | 8.0 | 80 | EL-01 | 1.45 | 129.0 | 11.2 | -0.0399 |
| 13 | 2.8 | 2.1 | 170 | EL-01 | 0.05 | 28.7 | 1.8 | -0.0043 |
| 14 | 8.2 | 8.0 | 170 | EL-01 | 0.48 | 10.4 | 45.7 | -0.0194 |
| 15 | 8.2 | 8.0 | 80 | EL-01 | 3.03 | 70.7 | 42.9 | -0.0399 |
| 16 | 8.2 | 2.1 | 170 | EL-01 | 0.38 | 22.6 | 17.0 | -0.0155 |
| 17 | 2.8 | 2.1 | 170 | FE-01 | 0.03 | 68.9 | 0.5 | -0.0032 |
| 18 | 5.5 | 5.1 | 125 | FE-01 | 1.06 | 55.3 | 19.1 | -0.0411 |
| 19 | 8.2 | 8.0 | 80 | FE-01 | 1.80 | 83.0 | 21.7 | -0.0795 |
| 20 | 8.2 | 2.1 | 50 | EL-01 | 0.49 | 9.9 | 49.2 | -0.0260 |
| 21 | 5.5 | 5.1 | 80 | EL-01 | 1.83 | 49.0 | 37.3 | -0.0536 |
| 22 | 8.2 | 2.1 | 170 | FE-01 | 0.78 | 44.3 | 17.5 | -0.0277 |
| 23 | 5.5 | 5.1 | 125 | EL-01 | 0.85 | 44.4 | 19.1 | -0.0384 |
| 24 | 5.5 | 5.1 | 200 | EL-01 | 0.04 | 46.5 | 0.8 | -0.0043 |
| 25 | 2.8 | 8.0 | 170 | FE-01 | 0.04 | 46.5 | 1.0 | -0.0040 |
| 26 | 5.5 | 5.1 | 200 | FE-01 | 0.15 | 52.7 | 2.9 | -0.0187 |
| 27 | 5.5 | 0.1 | 125 | EL-01 | 0.08 | 68.3 | 1.2 | -0.0043 |
| 28 | 5.5 | 5.1 | 125 | EL-01 | 0.60 | 37.6 | 16.0 | -0.0312 |
| 29 | 5.5 | 10.0 | 125 | FE-01 | 1.72 | 71.5 | 24.1 | -0.0499 |
| 30 | 5.5 | 5.1 | 125 | FE-01 | 0.55 | 31.0 | 17.6 | -0.0226 |
| 31 | 5.5 | 10.0 | 125 | EL-01 | 0.94 | 31.5 | 29.9 | -0.0268 |
| 32 | 10.0 | 5.1 | 80 | FE-01 | 2.22 | 104.6 | 21.2 | -0.0479 |
| 33 | 5.5 | 5.1 | 50 | FE-01 | 1.20 | 25.7 | 46.7 | -0.0558 |
| 34 | 10.0 | 5.1 | 125 | EL-01 | 1.33 | 30.3 | 44.0 | -0.0330 |
| 35 | 5.5 | 5.1 | 125 | FE-01 | 1.03 | 47.3 | 21.8 | -0.0335 |
| 36 | 5.5 | 5.1 | 125 | FE-01 | 0.91 | 55.2 | 16.4 | -0.0361 |
| 37 | 8.2 | 5.1 | 50 | EL-01 | 1.34 | 37.2 | 36.0 | -0.0384 |
| 38 | 10.0 | 0.1 | 200 | FE-01 | 0.26 | 35.8 | 7.4 | -0.0106 |
| 39 | 10.0 | 10.0 | 200 | FE-01 | 0.53 | 47.6 | 11.2 | -0.0324 |
| 40 | 5.5 | 10.0 | 50 | EL-01 | 0.92 | 74.4 | 12.3 | -0.0655 |
| 41 | 8.2 | 5.1 | 80 | EL-01 | 1.39 | 19.3 | 71.9 | -0.0439 |
| 42 | 10.0 | 10.0 | 170 | FE-01 | 0.57 | 29.4 | 19.5 | -0.0272 |
| 43 | 1.0 | 0.1 | 125 | EL-01 | 0.05 | 158.8 | 0.3 | -0.0035 |
| 44 | 1.0 | 10.0 | 125 | EL-01 | 0.07 | 82.8 | 0.8 | -0.0058 |
| 45 | 10.0 | 0.1 | 125 | FE-01 | 0.31 | 17.7 | 17.6 | -0.0205 |
| 46 | 8.2 | 5.1 | 170 | EL-01 | 0.32 | 20.2 | 15.7 | -0.0219 |
| 47 | 8.2 | 5.1 | 170 | EL-01 | 0.28 | 26.2 | 10.8 | -0.0414 |
| 48 | 10.0 | 2.1 | 80 | EL-01 | 1.75 | 28.4 | 61.5 | -0.0418 |

**Table S2**. XPS data of fouled samples. Given are the data of the top site *before* and *after* the first fouling and self-cleaning cycle.

| **sample** | **elemental composition / at%** | | | | | | **elemental ratio / %** | | | |
|---|---|---|---|---|---|---|---|---|---|---|
| | **C** | **F** | **O** | **N** | **S** | **Si** | **F/C** | **O/C** | **N/C** | **S/C** |
| PVDF-Ref (before) | 59.18 ±0.57 | 37.21 ±1.05 | 3.13 ±0.40 | 0.00 ±0.00 | 0.00 ±0.00 | 0.49 ±0.10 | 62.89 ±2.38 | 5.29 ±0.62 | 0.00 ±0.00 | 0.00 ±0.00 |
| PVDF-Ref (after) | 81.69 ±1.20 | 4.22 ±0.64 | 12.07 ±0.60 | 0.04 ±0.08 | 0.06 ±0.13 | 1.94 ±0.35 | 5.17 ±0.83 | 14.78 ±0.90 | 0.04 ±0.09 | 0.08 ±0.15 |
| PVDF-*g*-EL (before) | 61.51 ±0.47 | 32.27 ±1.32 | 5.03 ±0.67 | 0.58 ±0.10 | 0.00 ±0.00 | 0.62 ±0.36 | 52.47 ±2.50 | 8.17 ±1.04 | 0.93 ±0.15 | 0.00 ±0.00 |
| PVDF-*g*-EL (after) | 70.98 ±1.05 | 20.43 ±1.73 | 6.65 ±0.81 | 0.66 ±0.05 | 0.07 ±0.10 | 1.22 ±0.19 | 28.82 ±2.87 | 9.36 ±1.04 | 0.92 ±0.08 | 0.09 ±0.14 |
